# Nightlife and low immunity drove transmission of SARS-CoV-2 gamma in Luxembourg, 2021

**DOI:** 10.1038/s41598-025-94323-4

**Published:** 2025-03-25

**Authors:** Yolanda Pires Afonso, Dritan Bejko, Corinna Ernst, Conny Huberty, Anke Wienecke-Baldacchino, Sibel Berger, Malte Herold, Cécile Walczak, Leslie Ogorzaly, Anne Vergison, Joël Mossong

**Affiliations:** 1 Health Directorate, Luxembourg, Luxembourg; 2 Department of Microbiology, National Health Laboratory, Dudelange, Luxembourg; 3 Luxembourg Institute of Science and Technology, Esch-sur-Alzette, Luxembourg

**Keywords:** Disease prevention, Health policy, Microbiology

## Abstract

In summer 2021, while the Delta variant was predominant in many European countries, Luxembourg experienced a large outbreak of the P.1.17.1 SARS-CoV-2 Gamma variant. This surge coincided with the introduction of digital COVID-19 certificates and eased restrictions prior to national holiday festivities. We investigated the molecular and epidemiological factors contributing to SARS-CoV-2 Gamma transmission. All laboratory SARS-CoV-2 confirmed cases were interviewed by contact tracing teams on exposures at nightlife venues, family gatherings, schools, and workplaces. Gamma sublineages were investigated through environmental wastewater surveillance, in addition to whole genome sequencing of clinical data. Infection clusters were analysed combining phylogenetic with epidemiological exposures. From June 22 to August 12, 2021, we identified 1,049 cases of the Gamma P.1.17.1 sublineage, representing 39.2% of all sequenced cases. Initially, nightlife was the most frequent source setting (49.1%) with young adults accounting for 92.3% of these cases. Later, transmission shifted towards family (30.4%) setting with a corresponding increase in the age distribution. The C26645T mutation in the M-protein was associated with attending specific nightclub venues. Notably, 96.8% of cases were in unvaccinated individuals, and while P.1.17.1 was also detected in neighboring countries, 75.6% of the sequences submitted to GISAID were from Luxembourg. The study suggests the role of nightlife and low population immunity in driving the summer surge of SARS-CoV-2 among young adults, with digital certificates showing minimal impact on transmission. Sequencing of clinical and wastewater samples in combination with contact tracing was crucial to inform the outbreak investigation.

**Supplementary Information:**

The online version contains supplementary material available at 10.1038/s41598-025-94323-4.

## Introduction

Severe acute respiratory syndrome coronavirus 2 (SARS-CoV-2) caused a worldwide pandemic with 771 million recognized infections and more than 6.9 million associated deaths by 8 November 2023^1^. High levels of infection rapidly led to the evolution of mutations that conferred selective advantages, including higher transmissibility^2–4^, disease severity^2^, and evasion of immunity acquired from previous infection or vaccination^2,4^.

In May 2021, the World Health Organization (WHO) began labelling variants of concern (VOCs) using letters of the Greek alphabet^5^. During the first half of 2021, major VOCs in wide circulation included the variant Alpha (B.1.1.7), initially detected in the United Kingdom^6^, Beta (B.1.351) detected in South Africa^7^, Gamma (P.1) detected in Brazil^8^ and Delta (B.1.617.2) initially found in India^9^. Global efforts to limit and fight the pandemic were greatly hampered by the emergence of these VOCs, causing multiple major waves of SARS-CoV-2 infections, resulting in elevated levels of morbidity and mortality rates^10–12^. While SARS-CoV-2 vaccines have greatly contributed to reductions in coronavirus disease (COVID-19) associated morbidity and mortality, non-pharmaceutical interventions (NPIs) remained important for mitigating the spread of SARS-CoV-2 outbreaks in multiple contexts^13^.

The VOC Gamma was first reported in Brazil in late 2020 and rapidly raised concerns about increased transmissibility in a region with high pre-existing immunity relative to its parental lineage B.1.1.28^8,14^. Gamma was declared a VOC by the WHO on January 11, 2021 ^15^. The VOC Gamma possesses the triple mutational combination N501Y, E484K and K417T within its receptor binding domain (RBD) region, which greatly decreases sensitivity to the immune response in vaccine-induced and convalescent sera^3,16^. Additionally, some studies identified an increased risk of hospitalisation among younger adults^17,18^.

In Luxembourg, during the early months of 2021, significant social restrictions were implemented to minimise the spread of SARS-CoV-2^19^. As the COVID-19 vaccination program progressed and incidence rates fell by the summer of 2021, the national government gradually began easing COVID-19 restrictions. Restrictions included limitations on the number of people that could be invited at home, reduced opening times for restaurants and bars, and restrictions on seating at tables. The use of facemasks was also required in public indoor environments such as shops and workplaces.

On June 13, 2021, digital COVID-19 certificates (DCCs) - CovidCheck passports - were introduced. These allowed individuals who had either a full COVID-19 vaccination schedule, a recent negative COVID-19 test result within 48 h, or recovery from COVID-19 infection within 6 months to participate in events and indoor gatherings without wearing a facemask or maintaining a physical distance of 1.5 m. In addition to the DCCs, restaurants and bars could also offer rapid antigen tests on-site allowing the entry of customers who tested negative. This coincided with the end of curfew for the general population and the reopening of nightlife venues, including bars and nightclubs (detailed information available in the supplementary information). One week later, on the evening of June 22 (week 25), the country celebrates national holiday festivities in the capital with large public gatherings. Shortly after, an increase in cases with the Gamma VOC genotype was detected by the national surveillance system.

The present study investigates the molecular epidemiology of P.1.17.1 Gamma SARS-CoV-2 outbreak with environmental wastewater investigation in the summer of 2021 in Luxembourg. We combine epidemiological exposure data with phylogenetics to describe the settings where much of the transmission occurred. We compare our data with full-genome P.1.17.1 Gamma SARS-CoV-2 sequences submitted to GISAID from other countries around the same time period.

## Results

### SARS-CoV-2 variants circulation in Luxembourg 2021

**Fig. 1:**
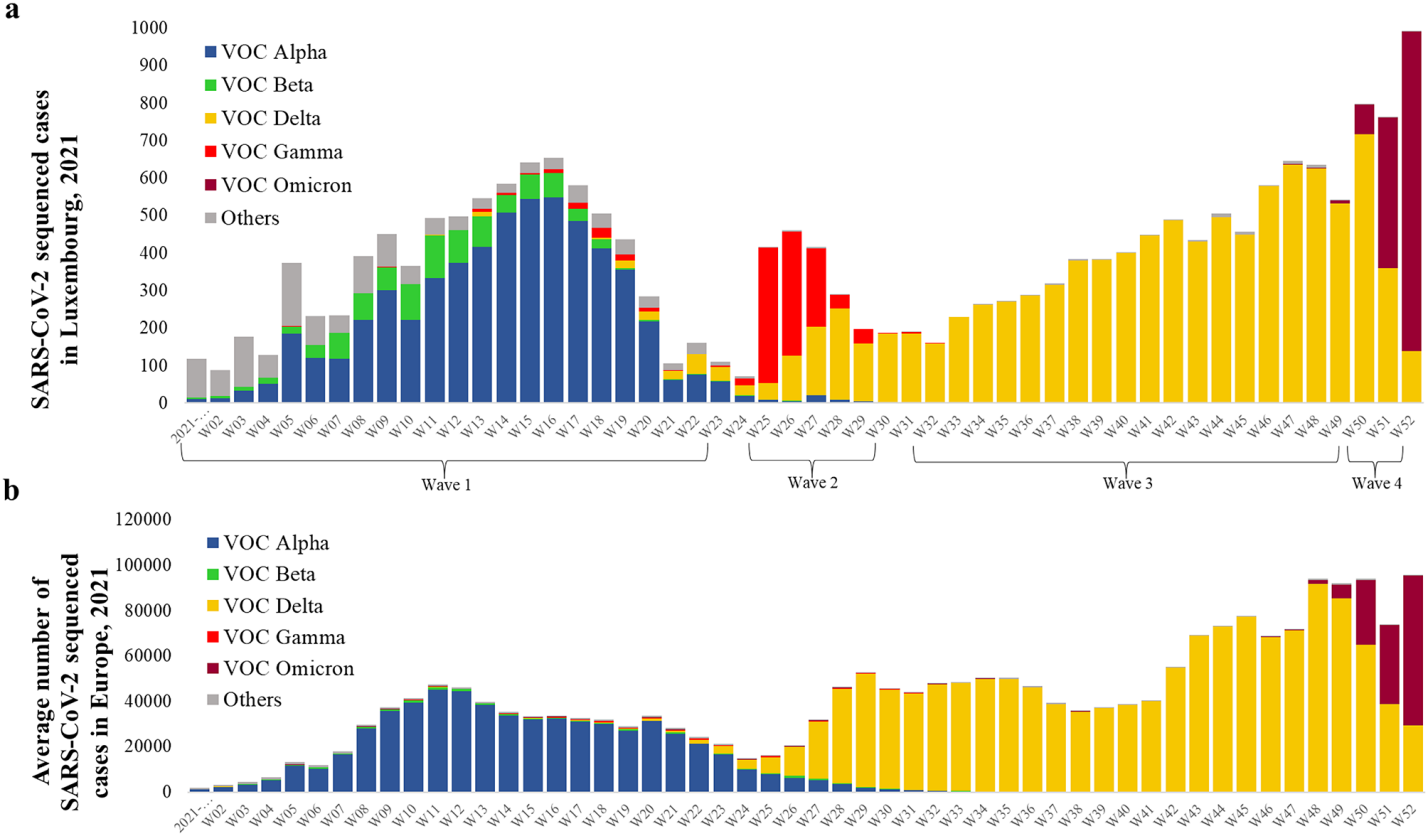
Temporal distribution of SARS-CoV-2 variants of concern (VOC) sequenced in Luxembourg and Europe during 2021. (**A**) Weekly number of SARS-CoV-2 sequenced cases in Luxembourg in 2021, stratified by major variants of concern (VOC Alpha, Beta, Gamma, Delta, and Omicron) and variants of interest are assigned as Others. Waves of infections in Luxembourg are labeled (Wave 1–4) for reference. (**B**) Weekly average number of SARS-CoV-2 sequenced cases in Europe in 2021, based on aggregated data from 30 European countries obtained from the European Respiratory Virus Surveillance Summary (ERVISS) platform.

During the full year of 2021, a total of 75,812 SARS-CoV-2 cases were reported to the national surveillance system, of which 27,468 (36.2%) were sequenced and 18,175 (24.0%) high-quality genomes were submitted to GISAID (Fig. S1). This substantial sequencing effort showed that Luxembourg experienced distinct waves of different VOCs in 2021. Similarly, to what has been observed worldwide, Luxembourg has been challenged with five VOCs at different periods, which includes Alpha and Beta (wave 1), Gamma (wave 2), Delta (wave 3) and Omicron (wave 4) (Fig. 1).

Beyond individual introductions, a wider circulation of distinct VOC Gamma sublineages (P.1, P.1.1 and P.1.16 and P.1.17) was first detected in April 2021. This included some small transmission clusters in three secondary schools, as well as a private event attended by students from these same schools around the Easter break (02–18 April 2021) (Fig. S2). By early June 2021, these Gamma sublineages had disappeared from circulation and cases of the new VOC Delta variant had appeared at relatively low incidence levels (Fig. 1).

**Fig. 2:**
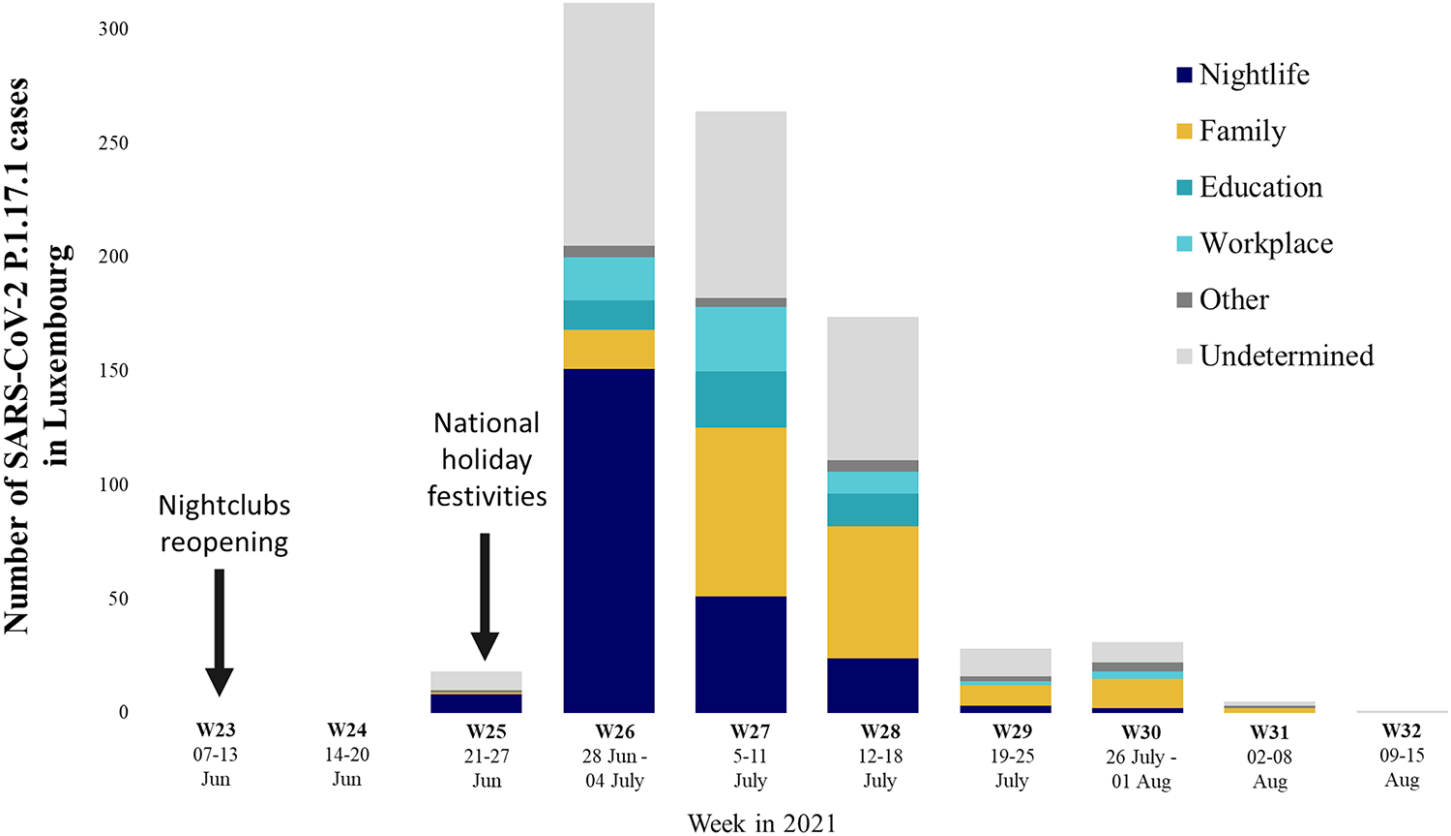
Epidemic curve depicting SARS-CoV-2 P.1.17.1 confirmed cases by date of sample collection and source of infection spanning from week 25 to week 32 (June-August) in Luxembourg 2021 (N = 826). The source of infection (e.g., nightlife, family, education, workplace, other and undetermined) is represented based on epidemiological data collected through contact tracing. The black arrows denote two events: nightclubs reopening on June 13 and national holiday festivities on June 22.

On June 13, 2021, the Luxembourgish government gradually lifted COVID-19 restrictions by introducing digital COVID-19 certificates (DCCs) in the hospitality sector. People could again meet and interact as usual, i.e., without facemasks and unrestricted gatherings at restaurants, bars, and nightlife venues as long as they had tested negative shortly before or possessed documented immunity through vaccination or prior infection. On June 22, 2021, one week after COVID-19 restrictions were relaxed, a large public gathering was held throughout the capital city as part of the national holiday celebrations, drawing significant crowds. The following week, the public surveillance system detected a rapid increase in COVID-19 cases, mainly among young people who reported attending the national holiday festivities (Fig. 2).

**Fig. 3:**
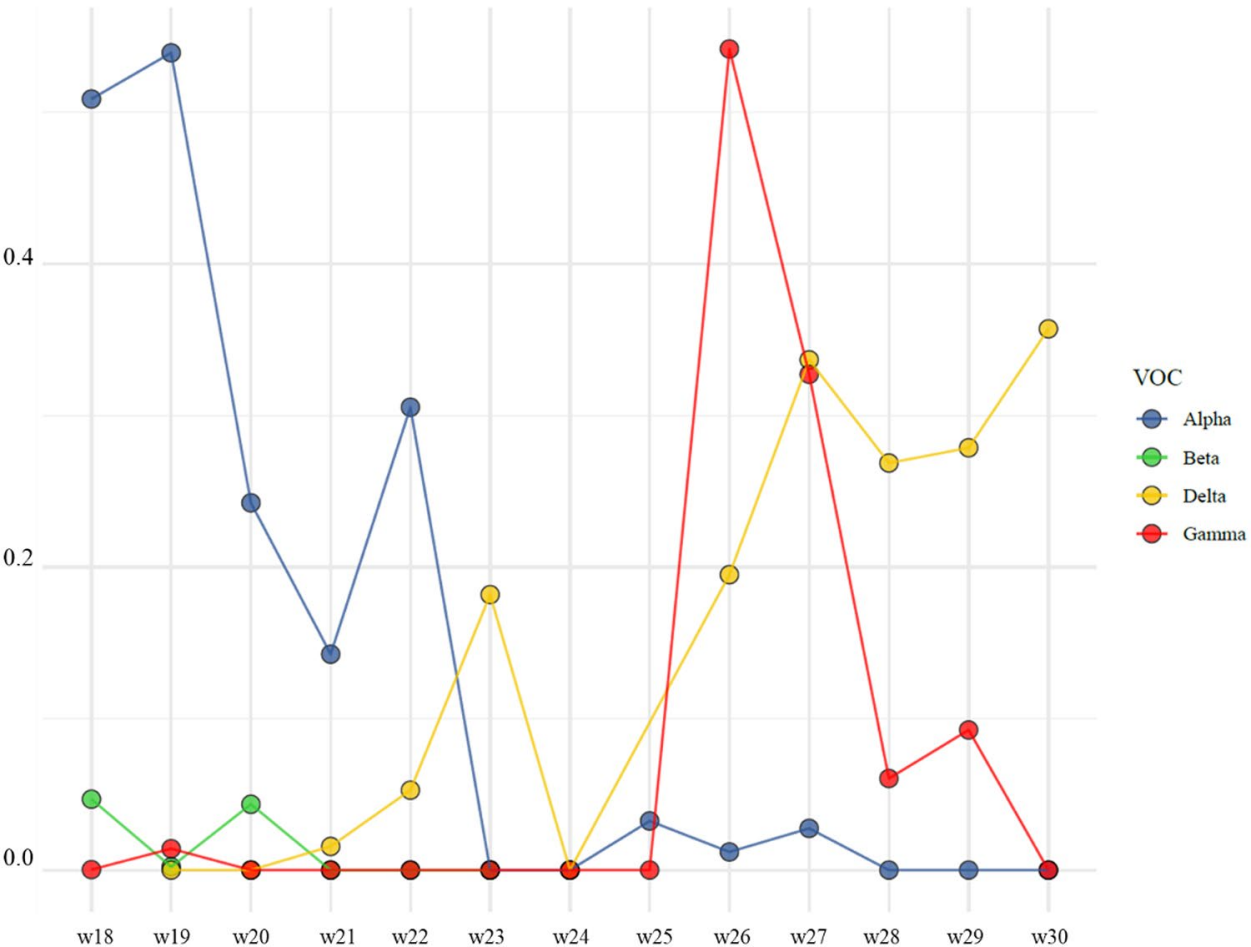
Fractional abundances of VOC-specific mutations in environmental wastewater samples. Samples during the timeframe 2021-05-01 to 2021-07-31 were selected and fractional abundances were aggregated by week across locations. Fractional abundances determined by RT-ddPCR are highlighted by coloured lines and points determined by levels of the following mutations: S: DEL69/70 (Alpha), S: K417N (Beta), S: D950N (Delta) and S: K417T (Gamma).

Genome sequencing revealed that most of the positive cases belonged to a particular Gamma P.1.17.1 sublineage (Fig. 3), which had not been detected before in Luxembourg, nor in Europe. The epidemic curve shows the peak in cases occurring during the week immediately following the national holiday festivities suggestive of one or several superspreading events. In total, 1,049 genetic sequences with the Gamma P.1.17.1 sublineage were identified, spanning the period between June 22 and August 15, 2021.

The increase in Gamma P.1.17.1 sublineage is also corroborated by environmental wastewater data, where a spike in the abundance of the Gamma specific mutation S: K417T up to 50% was observed in week 26 (Fig. 3). Whole genome sequencing of wastewater treatment plan (WWTP) samples reveals a similar picture, of which approximately 60% of the SARS-CoV-2 sequencing data from the WWTP can be associated to Gamma sublineages. Notably, high levels of P.1.17 sublineage were detected with a substantial portion assigned to P.1.17.1 sublineage in July 2021 (weeks 27–28) (Fig.S3).

For the current study, we investigated 826 cases (78.7%) of the Gamma P.1.17.1 sublineage for which epidemiological exposure data was collected by the contact tracing teams (Fig. S4). In the first phase of the outbreak (week 25–26), nightlife represented 49.1% of the most frequent source of infection, followed by family and workplace (5.2%) and education settings (3.7%) (Fig. 2). More than 90% of the cases were young adults with 214 (65.6%) cases between 15 and 29 and 87 (26.7%) cases among 30–34 years old (Table S1). Of the infected cases, 542 (65.6%) were not vaccinated and 250 (31.2%) were partially vaccinated. Only 26 (3.1%) had completed the full vaccination cycle (Table S1).

In the second phase of the outbreak (weeks 27–32), the proportion of cases increased among individuals aged 45–59 and 60 + years old, rising from 14 (4.3%) to 62 (12.4%) and from 2 (0.6%) to 24 (4.8%), respectively (Table S1). The relative risk of nightlife as a source of infection compared to family was high in the first phase (RRR: 7.89, CI: 3.63–17.12), while decreasing significantly in the second phase of the outbreak (RRR: 0.06, CI: 0.03–0.11) (Table 1).

**Table 1:** Multinomial logistic regression model of sources of infection (*N* = 826 cases).

	Nightlife		Education		Workplace		Travel		Other		Undetermined	
	RRR	CI	RRR	CI	RRR	CI	RRR	CI	RRR	CI	RRR	CI
Intercept	7.89***	3.63–17.12	3.15	0.99–9.97	0.27*	0.09–0.83	0.13*	0.02–0.71	0.03**	0.00-0.41	2.69**	1.31–5.52
Outbreak phase: W27-W32	0.06***	0.03–0.11	0.27**	0.11–0.63	0.25***	0.11–0.53	0.24*	0.07–0.79	0.9	0.11–7.63	0.16***	0.09–0.28
Sex: Male	1.30	0.84–2.01	1.45	0.74–2.83	3.02**	1.56–5.83	1.33	0.49–3.65	0.89	0.26–3.05	1.28	0.86–1.90
Age (cont.)	0.99	0.97-1.00	0.95**	0.91–0.98	1.01	0.99–1.04	1.01	0.97–1.05	1.02	0.98–1.07	1.02*	1.00-1.04
C26645T mutation: Yes	1.62*	1.04–2.52	0.39*	0.18–0.88	1.47	0.79–2.74	1.7	0.61–4.73	0.34	0.07–1.64	1.34	0.89-2.00
Fully vaccinated	0.43	0.05–3.84	0.00***	0.00–0.00	1.47	0.32–6.83	3.82	0.43–34.11	1.56	0.12–19.94	0.66	0.22–1.99
Partially vaccinated	2.26**	1.38–3.68	1.47	0.67–3.23	1.43	0.71–2.80	2.09	0.69–6.32	2.84	0.78–10.38	1.43	0.91–2.26

Reference source of infection: family setting.

RRR: relative risk ratio, CI: confidence interval, P value: *** <0.001, ** <0.01, *<0.05.

The transition into the second phase of the outbreak significantly affected all sources of infection compared to family, except for “other” sources of infection. Interestingly, males had a threefold higher relative risk than females of having “workplace” as their source of infection compared to “family” (RRR: 3.02, CI: 1.56–5.83). The odds of having a particular source of infection did not vary as a result of being fully or partially vaccinated (Table 1).

A logistic regression model examined the likelihood of hospitalisation across the outbreak phases. The relative risk ratio of hospitalisation was significantly higher for males (RRR: 2.7, CI: 1.05–6.95) and increased with age (RRR: 1.1, CI: 1.07–1.13) (Table S2), regardless of the vaccination status. No deaths were recorded among hospitalised cases.

### The role of National holiday festivities and certain nightlife venues

**Fig. 4:**
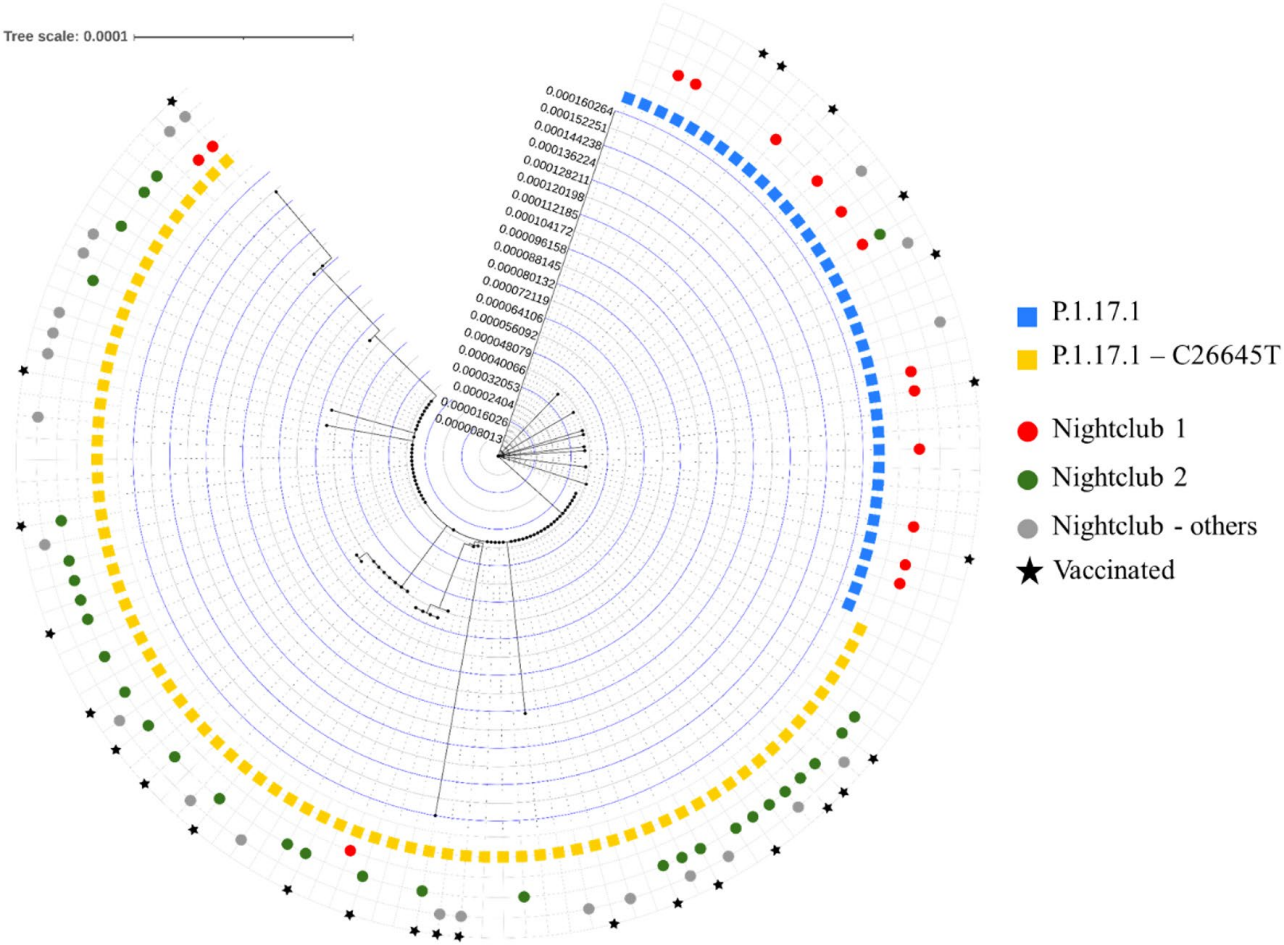
Phylogenetic tree of SARS-CoV-2 VOC Gamma P.1.17.1 sublineages cases who participated in national holiday festivities in Luxembourg city on June 22, 2021, linked to COVID-19 surveillance epidemiological data (N = 114). Relative distribution of P.1.17.1 (blue square) and P.1.17.1-C26645T (yellow square) strains at distinct nightclub venues (red for Nightclub 1, green for Nightclub 2, and grey for other nightclubs) among the 114 cases in Luxembourg is illustrated. The scale bar in the phylogenetic tree indicates the substitutions per site that corresponds to the length of branches in the tree.

In the first phase of the outbreak (week 25–26), 97 (68.8%) cases reporting nightlife as the source of infection were participating in the national holiday festivities. Of these, 58 (59.8%) aged between 15 and 59 years old, reported attending one or more nightclub venues. Integrating epidemiological data with WGS data, we identified two P.1.17.1 phylogenetic clusters, differentiated by the presence or absence of the C26645T mutation (N41N) in the M protein, among individuals who participated in national holiday festivities or attended nightclubs (Fig. 4, Fig. S5a). Contact tracing identified 16 different bars/nightclubs during this time, most located in the capital city.

A logistic regression model was applied to assess how different variables (e.g., nightclub attendance, age, vaccination status) influence the odds of acquiring the C26645T mutation in Luxembourg (Table S3). Here, we found that the likelihood of getting the C26645T mutation was significantly lower at nightclub 1 (RRR: 0.12, CI: 0.04–0.34) and significantly higher in nightclub 2 (RRR: 9.99, CI: 3.28–30.41), compared to not being to nightclubs at all (Table S3). Participation in national holiday festivities, as well as sex, age, and vaccination status, did not affect the odds of acquiring the mutation.

Overall, both P.1.17.1 and P.1.17.1-C26645T strains mainly infected young adults, in particular those aged between 15 and 29 and 30–44 years old (Fig. S5b). The C26645T mutation was observed in one wastewater sample in week 27 with an allele frequency of 97%. Just before the outbreak in week 23, COVID-19 vaccination coverage in younger age groups was relatively low, with 3% coverage in the 15–29 and 7% in the 30–44 age groups. Most P.1.17.1 cases (89.6%) were symptomatic.

### Circulation of P.1.17.1 elsewhere in Europe and the Americas

**Fig. 5:**
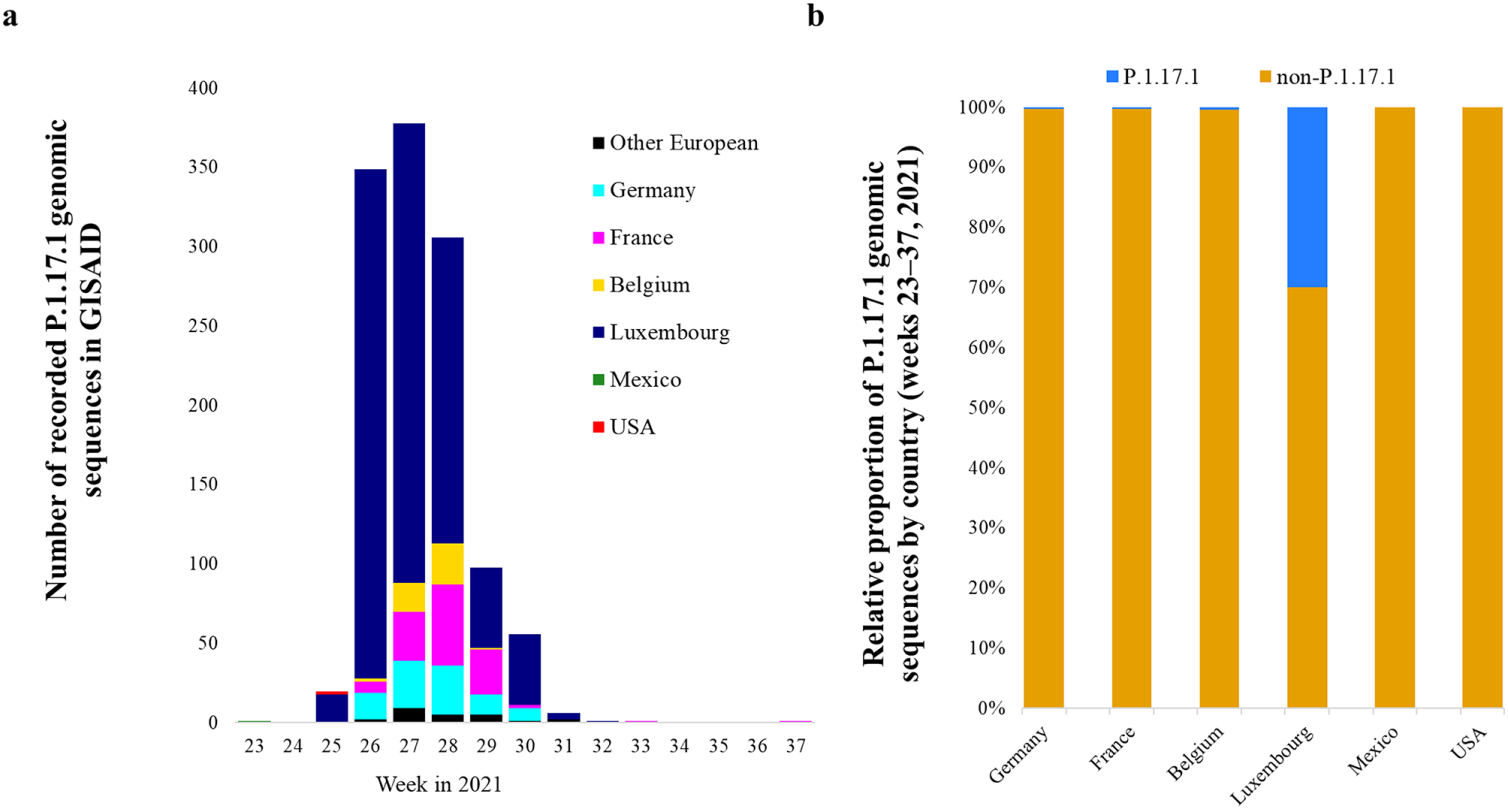
Epidemic curve and relative proportions of Gamma P.1.17.1 viral genomes submitted to GISAID (N = 1 222). (**A**) Weekly number of P.1.17.1 viral genomes recorded in non-European (*N* = 3, e.g., Mexico and USA) and European countries (*N* = 1219, e.g., Belgium, Denmark, France, Germany, Lithuania, Luxembourg, Mexico, Netherlands, Northern Ireland, Poland, Portugal, Spain, Switzerland) per collection date (weeks) retrieved from GISAID. (**B**) Relative proportion of P.1.17.1 genomic sequences compared to non-P.1.17.1 sequences in selected countries (Germany, France, Belgium, Luxembourg, Mexico, and USA) during weeks 23–37, 2021.

We analysed the global distribution and evolution of the Gamma P.1.17.1 sublineage, characterised by 12 mutations in the spike protein, including S: H49Y (Fig. S5a). GISAID contains a total of 1,222 genomic sequences of P.1.17.1 with sampling dates between June 8 (week 23) and September 15, 2021 (week 37) detected in 15 countries^20^, with Luxembourg (*N* = 924) accounting for 75.6% of the total (Fig. 5a).

The neighbouring countries France (*N* = 125), Germany (*N* = 99) and Belgium (*N* = 47) also reported relevant frequencies of Gamma P.1.17.1 sublineage. To emphasize the unique situation in Luxembourg, the relative proportion of P.1.17.1 genomic sequences to non-P.1.17.1 sequences was analysed. Luxembourg exhibited a markedly higher relative proportion of P.1.17.1 sequences compared to neighboring countries (France, Germany, and Belgium) and non-European countries (Mexico and the USA), where the presence of P.1.17.1 was minimal or undetected during the same period (Fig. 5b).

Phylogenetic analysis revealed that the earliest P.1.17.1 genomes identified in the USA and Mexico, were closely related to those observed in Luxembourg (Fig. S6a). The first reported case in Luxembourg was a traveller from Mexico who developed symptoms on June 18, with confirmation of the infection on June 22. This suggests that P.1.17.1 was likely introduced to Europe from the Americas. The C26645T mutation has been identified in P.1.17.1 genomes across international borders, with a notably incidence of 80.8% (*N* = 79/99) in Germany as compared to 45.6% (*N* = 421/924) in Luxembourg, to 57.6% (*N* = 71/121) in France, and to 34.0% (*N* = 16/46) in Belgium (Fig. S6b). Both strains of P.1.17.1 with or without C26645T mutation showed an increasing reporting of identical genetic sequences, suggesting cross-border transmission in Europe until mid-September 2021.

## Discussion

Our study illustrates the emergence of a distinct and uncommon VOC Gamma sublineage in a largely unimmunized population in a context where preventive measures and mask mandates were relaxed, and test-to-enter policy was introduced. Similar outbreaks in nightlife or party settings have been reported previously^21–26^ and indoor gatherings represent a favourable environment for COVID-19 transmission where preventative measures can be difficult to enforce^27^.

In 2021, Luxembourg sequenced 36.38% of SARS-CoV-2 samples on average, which was lower than Denmark (68.08%) and Iceland (53.22%) at EE/EEA level. During the Gamma outbreak period (weeks 23–32), Luxembourg increased its sequencing coverage to 51.24%, but still lagged behind Denmark (80.21%), Iceland (92.67%), Sweden (64.67%), Slovenia (76.57%), and Austria (58.58%). Therefore, the higher detection of Gamma sequences in Luxembourg was not due to higher sequencing effort or capacity relative to other countries.

Nightclubs in particular have been identified as high-risk environments for SARS-CoV-2 transmission due to their persistent overcrowding, the need to talk loudly, and inadequate ventilation^28,29^. Other studies have linked alcohol consumption to lower physical distance compliance^30–32^, and participants who often drank in a bar or nightclub had almost three times the odds of a COVID-19 diagnosis^30^. Age has been identified as a risk factor for SARS-CoV-2 infection in young adults attending bars and nightclubs compared to older adults^33^. Due to age prioritisation in the vaccination rollout campaign in Luxembourg, young adults remained largely unvaccinated, thus constituting a large susceptible group to the rapid spread in high-risk settings. At the time of the outbreak, COVID-19 vaccination coverage in younger age groups was too low to provide protection against wider transmission. Together, our findings highlight the role of nightlife settings as a conduit for superspreading events among unvaccinated young adults leading to subsequent dispersal within the wider more vulnerable community^29^.

The outbreak occurred in the context of digital COVID certificates that were implemented in Luxembourg and in other countries^34^ to facilitate safe access to hospitality venues and encourage vaccination uptake, especially among young people. However, the ethical and political implications of DCCs have been debated, and their effect on uptake seems to be limited to countries with low vaccination coverage^35^. Moreover, the impact of DCCs on transmission is questionable when a large proportion of the target population is not immune and relies on testing. While rapid antigen testing may detect asymptomatic cases with high viral loads, their sensitivity is likely to be too low to detect all viral shedders. Several factors will influence the sensitivity and the false negativity rate, like the viral load, the prevalence of the disease in the population, and whether the case is symptomatic or not. A Cochrane review estimated the false negativity rate among symptomatic people during the first week after symptoms onset to be around 20% if the prevalence of the disease was 5% ^36^. However, at a prevalence of 0.5% among asymptomatic people, the false negativity rate would be between 33% and 52% ^37^. We think the second scenario applies to the described situation as the estimated prevalence was less than 0.5% and that asymptomatic people would more likely participate in nightlife activities and will request an onsite antigen test to participate in indoor venues. Our study and others^21^ have shown that DCCs failed to prevent major outbreaks in hospitality settings.

Our genomic analysis suggests that the VOC Gamma P.1.17.1 sublineage observed in our outbreak was not circulating in Luxembourg or Europe before June 2021, but was probably introduced locally by a traveller from the Americas around that time. The variant did not persist beyond mid-September and did not spread widely in other countries, indicating that it did not have a higher transmission potential or fitness advantage compared to the VOC Delta variant, which became globally dominant^21,37,38^. Structural studies have shown that H49Y mutation in the spike protein of the VOC Gamma P.1.17.1 sublineage has a higher affinity to the ACE2 receptor than the D614G mutation and the wild-type protein, which may facilitate virus cell entry^39,40^. However, epidemiological factors, such as superspreading events may have contributed more to the temporary high local prevalence of this variant. Thus, the emergence and temporary predominance of Gamma P.1.17.1 was likely facilitated by the very low incidence prior to the outbreak.

Luxembourg was a major hub for cross-border transmission of the P.1.17.1 sublineage to neighbouring France, Germany, and Belgium until mid-September 2021. The C26645T mutation, which occurred in 49% of P.1.17.1 genomes worldwide has also been detected in several other lineages, including B.1.351.5 (95%), B.1.428.3 (88%) and B.1.428.2 (79%), indicating that it is not unique to the P.1.17.1 sublineage^20^. The distribution of the C26645T exemplifies how individual mutations can be leveraged for epidemiological outbreak characterization beyond the sublineage level. We have shown 60% of SARS-CoV-2 sequencing data can be associated to Gamma sublineages, as independent factor of sequencing efforts during this time. Even with low levels of circulating virus, sequencing data derived from wastewater sampling could have predicted the increase in VOC Gamma including identification of circulating mutations. However, increased sampling density would have been required to trace the outbreak by wastewater epidemiology on a level of detail comparable to what was observed in the clinical sequencing data. SARS-CoV-2 incidence is usually lower in summer in the general population than in other seasons. Given the relatively low numbers of cases in the weeks before the Gamma VOC outbreak, partly due to the COVID-19 restrictions in place contributing to reduce community-based transmission, there may be a limited immunity from previous infections. In addition, there is still a risk of local transmission arising in low prevalence settings initiated by importations from elsewhere. Overall, the combination of low population immunity with nightlife activities contributed to the local transmission of Gamma SARS-CoV-2, particularly among young adults who were largely unvaccinated at this time, due to the prioritisation phases of the COVID-19 vaccination campaign.

Our study shows that indoor summer-related activities among young non-immune people may facilitate the emergence and spread of respiratory viruses. Seasonality may affect the transmission dynamics of respiratory viruses through variations in temperature, humidity, sunlight, and human behaviour. For instance, human behaviour may change seasonally, such as increased indoor activities, social gatherings, or travel. However, the role of seasonality in SARS-CoV-2 transmission is unclear and may be modulated by other factors such as population immunity, public health measures and viral variants.

Our study has several limitations. The epidemiological investigation relied on phone interviews and voluntary cooperation of cases, which might have missed some contacts or left them untested, obscuring additional exposure information and transmission chains in the community. The potential for multiple exposures in different settings also complicates analyses. Moreover, many cases may have escaped detection, either due to a high percentage of cross-border commuters testing abroad or to the presence of asymptomatic infections. While wastewater surveillance data provided an unbiased snapshot of the viral diversity circulating in the general population within Luxemburg, unaffected by individuals’ health-seeking behaviours, inferring direct transmission chains from WWTPs between countries is rarely feasible due to the lack of comprehensive wastewater-based epidemiology surveillance data. The accuracy of international transmission was also limited by the availability and timing of sequenced cases in GISAID. More comprehensive data on travel history, source of infection, symptoms and clinical outcomes could enhance the utility of genomic epidemiology.

Our study shows how genomic sequencing and epidemiological data can identify infection clusters and exposure settings of SARS-CoV-2 variants. We focused on a large P.1.17.1 outbreak that occurred in the country between mid-June and August 2021, when COVID-19 measures were relaxed, and population immunity was low. Our data suggests that the lineage P.1.17.1 was likely introduced to Europe from the Americas and then was mainly transmitted among unvaccinated young adults during nightlife activities. Our study illustrates the importance of combining genomic surveillance and contact tracing to identify high-risk transmission settings and inform outbreak investigations and prevention strategies. Genomic surveillance has the potential to create an abundant source of information, enabling the monitoring of pathogen transmission and evolution at local, regional, and global scales.

## Methods

### Study design

This study is a retrospective investigation of SARS-CoV-2 cases occurring in Luxembourg focusing on the summer period from June 22 (week 25) until August 15, 2021 (week 32). Our analysis is based on confirmed PCR positive cases of SARS-CoV-2 that were notified on a mandatory basis by clinical laboratories and automatically integrated into the contact tracing system of the Health Directorate.

The P.1.17.1 outbreak was divided into two phases for a detailed investigation of Gamma infection dynamics over its course. The first phase corresponding to the onset of the outbreak covers calendar weeks 25 and 26, while the second phase from weeks 27 to 32 is characterised by the subsequent transmission of Gamma infections within the population. Epidemiological data on individuals’ occupations, workplace or school attendance, household and familial contacts, leisure activities including exposure to bars, restaurants, and nightlife venues, travel history, as well as vaccination status were collected. This data was collected by the Health Authorities in Luxembourg in strict accordance with COVID-19 law^41^ applicable during the period of the pandemic. The COVID-19 law requires both cases and contacts to report the circumstances and dates of their contacts, going back at least 48 h before a positive test or symptom onset. Informed consent for clinical and epidemiological data was obtained when individuals were contacted by the contact tracing teams. All cases were automatically sent, by SMS or email, a seven-day isolation certificate that also ensured a full reimbursement of sick leave by the national health insurance.

Contact with a known positive case within 14 days of the incubation period was considered the probable source of infection. We categorised sources as family, workplace/ education, nightlife, and others (e.g., travel, sports) if the case reported prior contact with a known positive case in any of these settings. The source was unknown if no contact with a positive case or multiple settings were reported.

### Whole-genome sequencing (WGS)

Clinical laboratory specimens that were positive for SARS-CoV-2 RNA were sent to the National Health Laboratory for sequencing. Samples containing at least one CT value below 35 were eligible to be sequenced and whenever possible to those cases reporting nightlife activities exposure during the time of this study. Sequencing was performed using an amplicon approach, based on an in-house primer scheme. The primer scheme results in 51 overlapping amplicons with an approximate length of 900 bp and an overlap of 200 bp. Library preparation was done using the Illumina DNA prep kit, following the manufacturer’s instructions. Per run, 192 samples were sequenced according to a 151 bp-paired end approach and indexed with the IDT-ILMN Nextera DNA UD Indexes 384—Nextera DNA Flex set on an Illumina instrument (either MiniSeq^®^ or MiSeqDX^®^ (San Diego, CA, USA)). Pre-analytical steps and subsequent reference mapping-based consensus-sequence generation were performed as described by Ernst et al. 2023^42^. High-quality sequences with greater than 90% sequencing coverage were submitted to the Global Initiative on Sharing All Influenza Data (GISAID).

Following WGS, lineage information was transmitted on a weekly basis to the Health Directorate and integrated with available epidemiological exposure information.

### Detection and sequencing in wastewater

From May 01 to July 31, 2021, wastewater samples were collected at the inlet of the wastewater treatment plant over a 24-hour period using automatic samplers (Teledyne ISCO, Lincoln, NE, USA) for 11 locations at a mean interval of 4.5 days (RT-ddPCR) or three locations in weekly intervals (whole genome sequencing), respectively. Viral RNA extraction was done with the easyMAG (Biomérieux) according to the manufacturer’s recommendations. Two hundred µl of each sample was placed in the disposable sample vessel and the sample vessel was loaded onto the extractor. Viral RNA was manually extracted from 140 µL of sample concentrates using the QiAamp Viral RNA mini kit (Qiagen, Hilden, Germany) into a final volume of 60 µL according to the manufacturer’s protocol. Samples were transported and processed as described previously^43^. In brief, ultrafiltration using an Amicon Plus-15 10 kDa (Millipore) device (3,220 x g, 25 min, 4 °C) was performed after the removal of larger particles by centrifugation (2.400 x g, 20 min). SARS-CoV-2 RNA was assessed by RT-digital droplet PCR (RT-ddPCR) to quantify characteristic VOC mutations and the wild type (WIV04/2019, WT) sequence in one single tube multiplex mutation assay designed by BioRad. Samples were scanned using the QX200 system (BioRad) and analysed using the QuantaSoft-Analysis software (BioRad). For each sample, the number of negative and WT or Mut RT-ddPCR positive droplets were recorded and used to determine the WT or Mut concentrations (genome copies/reaction) and converted to genome copies/L of wastewater. Fractional abundances were averaged across locations weighted by population equivalents and samples were aggregated by calendar week of sampling. WGS data from wastewater samples were analysed as reported previously^43^ including the detection of mutations with LoFreq and the abundances of lineages were predicted with VLQ^44^ utilising all genomes from Luxembourg available on GISAID on April 18, 2023, as reference. Only samples with an average depth over 10 and at least 40% genome coverage are reported.

### Phylogenetic analysis

A phylogeny was constructed for samples with at least 95% sequencing coverage. Multisequence alignment of SARS-CoV-2 Gamma sequences was performed by mapping against the Wuhan-Hu-1 reference genome (NCBI Reference Sequence: NC_045512.2) using minimap2^45^ from the publicly available Pathogenwatch platform (version 19.3.0)^46^. The resulting phylogenetic tree was downloaded in newick format, annotated, and visualised on the web application Interactive Tree of Life (iTOL v6.6)^47^. A phylogenetic tree was constructed using P.1.17.1 Gamma SARS-CoV-2 genetic sequences with epidemiological link to national holidays attendance, independent of the source of infection (*N* = 114).

A time-resolved tree was performed using the Nextstrain analysis applying the ncov Nextstrain pipeline. Briefly, we first installed the nextstrain/augur environment and cloned the ncov GitHub repository (https://github.com/nextstrain/ncov). The SARS-CoV-2 genomic sequences and associated metadata for our samples were retrieved from GISAID and formatted as input for the Augur pipeline. A workflow file was created to point to our GISAID dataset (in tar format), and set the root to refine step to “oldest” to generate a time-resolved tree. All other parameters were kept at their default values as provided by the ncov-pipeline. The workflow was executed by running the Nextstrain build command within the Nextstrain environment, following the steps outlined in the official tutorial (https://docs.nextstrain.org/projects/ncov/en/latest/tutorial/example-data.html)^48^.

### Statistical analysis

The statistical analysis was performed on a sample of 826 individuals. The descriptive statistics of all relevant variables are included in supplementary Table 1. In addition, a sub-sample of 207 individuals was created, consisting of individuals for whom data were available regarding either participation in national holiday festivities or presence in nightclubs.

Individuals were considered fully vaccinated if the positive test was greater than or equal to 14 days after receiving the second dose or greater than 28 days from a single dose schedule of a complete first cycle. Likewise, individuals were considered partially vaccinated from the day of administration of the first dose while not being fully vaccinated, and as unvaccinated if no vaccination record was found. For the variable “presence in nightclub”, “other nightclubs” refers to those nightclubs where the total number of recorded COVID-19 cases was five or fewer.

In this study, we developed three models to investigate the relationship between phases of the outbreak, sources of infection and hospitalisations with the role of nightclubs in the spreading of the C26645T mutation. The first model, a multinomial logistic regression model, explores the association between the phase of the outbreak and the different sources of infection (the outcome), controlling for sex, age, presence of the C26645T mutation and vaccination status. In a multinomial logistic regression with $\:k=\text{1,2},\ldots,K$ outcomes where $\:K\:$is chosen as the reference outcome, we estimate $\:K-1$ binary logistic regressions on the form

$$\:\text{ln}\left(\frac{P\left({Y}_{i}=k\right)}{P\left({Y}_{i}=K\right)}\right)={\beta\:}_{k}\cdot \:{\mathbf{X}}_{i}\:,\:k<K$$

where $\:{\beta\:}_{k}$ is a vector of regression coefficients and $\:{\mathbf{X}}_{i}$ is a vector of independent variables. Results are reported in relative risk ratios (RRR), which are obtained by exponentiating the coefficients.

The second model investigated the likelihood of hospitalisation across outbreak phases, controlling for sex, age, presence of the C26645T mutation and vaccination status. Finally, the third model, performed on the sub-sample of 207 individuals, aimed to investigate the association between attendance at particular nightclubs and the occurrence of the C26645T mutation, controlling for sex, age, vaccination status and presence at national holiday festivities.

## Electronic supplementary material

Below is the link to the electronic supplementary material.

Supplementary Material 1

## Data Availability

The datasets generated and/or analysed during the current study are available in the GISAID repository under the code EPI_SET_231113gb (composed of 826 genomes, Fig. 2, 10.55876/gis8.231113gb) and EPI_SET_231113zv (composed of 1’222 individual genomes, Fig. 5, 10.55876/gis8.231113zv). The wastewater sequencing data in repository ENA under the BioProject ID PRJNA1212683 (https://www.ebi.ac.uk/ena/browser/view/PRJNA1212683). Supplementary methods, figures, and tables are provided.

## Abbreviations

COVID-19: Coronavirus disease 2019
DCCs: Digital COVID certificates, RT-ddPCR: RT-digital droplet PCR PCR: Polymerase chain reaction
SARS-CoV-2: Severe acute respiratory syndrome coronavirus 2
SNP: Single Nucleotide Polymorphism
VOC: Variant of Concern
WGS: Whole Genome Sequencing
WHO: World Health Organization

## References

[1] WHO. *WHO Coronavirus (COVID-19) Dashboard*. https://covid19.who.int/

[2] Campbell, F. et al. Increased transmissibility and global spread of SARS-CoV-2 variants of concern as at June 2021. *Eurosurveillance* **26** (2021). 10.2807/1560-7917.ES.2021.26.24.2100509 PMC8212592 34142653

[3] Cele, S. et al. Escape of SARS-CoV-2 501Y.V2 from neutralization by convalescent plasma. *Nature* **593**, 142–146 (2021). 33780970 10.1038/s41586-021-03471-w PMC9867906

[4] Funk, T. et al. Characteristics of SARS-CoV-2 variants of concern B.1.1.7, B.1.351 or P.1: data from seven EU/EEA countries, weeks 38/2020 to 10/202. *Eurosurveillance* **26**(16), 2100348 (2021). 10.2807/1560-7917.ES.2021.26.16.2100348 PMC8063589 33890566

[5] WHO. *WHO Announces Simple, Easy-to-Say Labels for SARS-CoV-2 Variants of Interest and Concern*. (2021). https://www.who.int/news/item/31-05-2021-who-announces-simple-easy-to-say-labels-for-sars-cov-2-variants-of-interest-and-concern

[6] Davies, N. G. et al. Estimated transmissibility and impact of SARS-CoV-2 lineage B.1.1.7 in England. *Science* **372**, eabg3055 (2021). 33658326 10.1126/science.abg3055 PMC8128288

[7] Tegally, H. et al. Detection of a SARS-CoV-2 variant of concern in South Africa. *Nature* **592**, 438–443 (2021). 33690265 10.1038/s41586-021-03402-9

[8] Faria, N. R. et al. Genomics and epidemiology of the P.1 SARS-CoV-2 lineage in Manaus, Brazil. *Science* **372**, 815–821 (2021). 33853970 10.1126/science.abh2644 PMC8139423

[9] Mlcochova, P. et al. SARS-CoV-2 B.1.617.2 delta variant replication and immune evasion. *Nature* **599**, 114–119 (2021). 34488225 10.1038/s41586-021-03944-y PMC8566220

[10] Grint, D. J. et al. Case fatality risk of the SARS-CoV-2 variant of concern B.1.1.7 in England, 16 November to 5 February. *Euro. Surveill Bull. Eur. Sur Mal Transm Eur. Commun. Dis. Bull.* **26**, 2100256 (2021). 10.2807/1560-7917.ES.2021.26.11.2100256 PMC7976383 33739254

[11] Stepanova, M. et al. The impact of variants and vaccination on the mortality and resource utilization of hospitalized patients with COVID-19. *BMC Infect. Dis.* **22**, 702 (2022). 35996076 10.1186/s12879-022-07657-z PMC9394045

[12] Nyberg, T. et al. A standardised protocol for relative SARS-CoV-2 variant severity assessment, applied to Omicron BA.1 and delta in six European countries, October 2021 to February 2022. *Euro. Surveill Bull. Eur. Sur Mal Transm Eur. Commun. Dis. Bull.* **28**, 2300048 (2023). 10.2807/1560-7917.ES.2023.28.36.2300048 PMC10486193 37676146

[13] Brauner, J. M. et al. Inferring the effectiveness of government interventions against COVID-19. *Science* **371**, eabd9338 (2021). 33323424 10.1126/science.abd9338 PMC7877495

[14] Naveca, F. G. COVID-19 in Amazonas, Brazil, was driven by the persistence of endemic lineages and P.1 emergence. *Nat. Med.* **27**, 1230–1238 (2021). 10.1038/s41591-021-01378-7 34035535

[15] WHO. *Tracking SARS-CoV-2 Variants*. https://www.who.int/en/activities/tracking-SARS-CoV-2-variants

[16] Gupta, R. K. & Will SARS-CoV-2 variants of concern affect the promise of vaccines? *Nat. Rev. Immunol.* **21**, 340–341 (2021). 33927376 10.1038/s41577-021-00556-5 PMC8082481

[17] Zavascki, A. P. et al. Evaluation of clinical course of gamma (P.1) variant of concern versus lineages in hospitalized patients with COVID-19 in a reference center in Brazil. *Am. J. Trop. Med. Hyg.* **107**, 245–251 (2022). 35895420 10.4269/ajtmh.21-1264 PMC9393469

[18] Nonaka, C. K. V. et al. SARS-CoV-2 variant of concern P.1 (Gamma) infection in young and middle-aged patients admitted to the intensive care units of a single hospital in Salvador, Northeast Brazil, February 2021. *Int. J. Infect. Dis.* **111**, 47–54 (2021). 34390857 10.1016/j.ijid.2021.08.003 PMC8356754

[19] Le Gouvernement du Grand-Duché de Luxembourg. *Version Consolidée Applicable Au 01/02/2021: Loi Du 17 Juillet 2020 Portant Introduction d’une Série de Mesures de Lutte Contre La Pandémie Covid-19*. (2021). https://legilux.public.lu/eli/etat/leg/loi/2020/07/17/a624/consolide/20210201

[20] Gangavarapu, K. *et al. P.1.17.1 Lineage Report*. https://outbreak.info/situation-reports?xmin=2022-07-17&xmax=2023-01-17&pango=P.1.17.1

[21] Koopsen, J. et al. Epidemiologic and genomic analysis of SARS-CoV-2 delta variant Superspreading event in nightclub, the Netherlands, June 2021. *Emerg. Infect. Dis.* **28**, 1012–1016 (2022). 35271792 10.3201/eid2805.212019 PMC9045423

[22] Muller, N. et al. Severe acute respiratory syndrome coronavirus 2 outbreak related to a nightclub, Germany, 2020. *Emerg. Infect. Dis.* **27**, 645–648 (2020). 33263514 10.3201/eid2702.204443 PMC7853558

[23] Kang, C. R. et al. Coronavirus disease exposure and spread from nightclubs, South Korea. *Emerg. Infect. Dis.* **26**, 2499–2501 (2020). 32633713 10.3201/eid2610.202573 PMC7510694

[24] Picard, G. Investigation de cas groupés d’infections à SARS-CoV-2 dans la station balnéaire de Quiberon, Bretagne, Juillet-Août 2020 / Investigation of cluster of SARS-CoV-2 infections in the seaside resort of quiberon, Brittany, France, July-August. (2020).

[25] Brandal, L. T. et al. Outbreak caused by the SARS-CoV-2 Omicron variant in Norway, November to December 2021. *Eurosurveillance* 26, (2021). 10.2807/1560-7917.ES.2021.26.50.2101147 PMC8728491 34915975

[26] van der Veer, B. M. J. W. et al. SARS-CoV-2 transmission dynamics in bars, restaurants, and nightclubs. *Front. Microbiol.* **14**, 1183877 (2023). 37275153 10.3389/fmicb.2023.1183877 PMC10232797

[27] Brown, C. M. et al. July. Outbreak of SARS-CoV-2 Infections, including COVID-19 Vaccine Breakthrough Infections, Associated with Large Public Gatherings — Barnstable County, Massachusetts, 70, (2021). (2021). 10.15585/mmwr.mm7031e2 PMC8367314 34351882

[28] Chau, N. V. V. et al. Superspreading event of SARS-CoV-2 infection at a bar, Ho Chi Minh City, Vietnam. *Emerg. Infect. Dis.* **27**, 310–314 (2021). 33063657 10.3201/eid2701.203480 PMC7774544

[29] Imamura, T. et al. Transmission of COVID-19 in nightlife, household, and health care settings in Tokyo, Japan, in 2020. *JAMA Netw. Open.* **6**, e230589 (2023). 36826818 10.1001/jamanetworkopen.2023.0589 PMC9958531

[30] Allen, H. K., Cohen-Winans, S., Armstrong, K., Clark, N. C. & Ford, M. A. COVID-19 exposure and diagnosis among college student drinkers: links to alcohol use behavior, motives, and context. *Transl Behav. Med.* **11**, 1348–1353 (2021). 34037226 10.1093/tbm/ibab059 PMC8194531

[31] Gurrieri, L., Fairbairn, C. E., Sayette, M. A. & Bosch, N. Alcohol narrows physical distance between strangers. *Proc. Natl. Acad. Sci.* 118, e2101937118 (2021). 10.1073/pnas.2101937118 PMC8157913 33972448

[32] Fitzgerald, N. et al. Managing COVID-19 transmission risks in bars: an interview and observation study. *J. Stud. Alcohol Drugs*. **82**, 42–54 (2021). 33573721

[33] Poletti, P. et al. Association of age with likelihood of developing symptoms and critical disease among close contacts exposed to patients with confirmed SARS-CoV-2 infection in Italy. *JAMA Netw. Open.* **4**, e211085 (2021). 33688964 10.1001/jamanetworkopen.2021.1085 PMC7948061

[34] Oliu-Barton, M. et al. The effect of COVID certificates on vaccine uptake, health outcomes, and the economy. *Nat. Commun.* **13**, 3942 (2022). 35803909 10.1038/s41467-022-31394-1 PMC9263819

[35] Mills, M. C. & Rüttenauer, T. The effect of mandatory COVID-19 certificates on vaccine uptake: synthetic-control modelling of six countries. *Lancet Public. Health*. **7**, e15–e22 (2022). 34914925 10.1016/S2468-2667(21)00273-5 PMC8668192

[36] Dinnes, J. et al. Rapid, point-of-care antigen tests for diagnosis of SARS-CoV-2 infection. *Cochrane Database Syst. Rev.* (2022). (2022). 10.1002/14651858.CD013705.pub3 PMC9305720 35866452

[37] Moen, L. V., Vollan, H. S., Bråte, J., Hungnes, O. & Bragstad, K. Molecular epidemiology of the Norwegian SARS-CoV-2 delta lineage AY.63. *Viruses* **14**, 2734 (2022). 36560738 10.3390/v14122734 PMC9781678

[38] Alizon, S. et al. Rapid spread of the SARS-CoV-2 Delta variant in some French regions, June 2021. *Eurosurveillance* 26, (2021). 10.2807/1560-7917.ES.2021.26.28.2100573 PMC8284044 34269174

[39] Bello, M., Hasan, M. K. & Hussain, N. Energetic and structural basis for the differences in infectivity between the wild-type and mutant Spike proteins of SARS-CoV-2 in the Mexican population. *J. Mol. Graph Model.* **107**, 107970 (2021). 34242876 10.1016/j.jmgm.2021.107970 PMC8258282

[40] Sixto-López, Y. et al. Structural insights into SARS-CoV-2 Spike protein and its natural mutants found in Mexican population. *Sci. Rep.* **11**, 4659 (2021). 33633229 10.1038/s41598-021-84053-8 PMC7907372

[41] Le Gouvernement du Grand-Duché de Luxembourg. *Version Consolidée Applicable Au 13/06/2021: Loi Du 17 Juillet 2020 Portant Introduction d’une Série de Mesures de Lutte Contre La Pandémie Covid-19*. (2021). https://legilux.public.lu/eli/etat/leg/loi/2020/07/17/a624/consolide/20210613

[42] Ernst, C. et al. A molecular and epidemiological investigation of a large SARS-CoV-2 outbreak in a Long-Term care facility in Luxembourg, 2021. *Geriatr. Basel Switz.* **8**, 19 (2023). 10.3390/geriatrics8010019 PMC9957261 36826361

[43] Herold, M. et al. Genome sequencing of SARS-CoV-2 allows monitoring of variants of concern through wastewater. *Water* **13**, 3018 (2021).

[44] Baaijens, J. A. et al. Lineage abundance Estimation for SARS-CoV-2 in wastewater using transcriptome quantification techniques. *Genome Biol.* **23**, 236 (2022). 36348471 10.1186/s13059-022-02805-9 PMC9643916

[45] Li, H. Minimap2: pairwise alignment for nucleotide sequences. *Bioinformatics* **34**, 3094–3100 (2018). 29750242 10.1093/bioinformatics/bty191 PMC6137996

[46] Pathogenwatch *A Global Platform for Genomic Surveillance.* https://pathogen.watch/.

[47] Letunic, I. & Bork, P. Interactive tree of life (iTOL) v5: an online tool for phylogenetic tree display and annotation. *Nucleic Acids Res.* **49**, W293–W296 (2021). 33885785 10.1093/nar/gkab301 PMC8265157

[48] Hadfield, J. et al. Nextstrain: real-time tracking of pathogen evolution. *Bioinforma Oxf. Engl.* **34**, 4121–4123 (2018). 10.1093/bioinformatics/bty407 PMC6247931 29790939

