# Supplementary material for: Nightlife and low immunity drove transmission of SARS-CoV-2 gamma in Luxembourg, 2021
Source: Sci Rep. 2025 Mar 25;15:10293. doi: 10.1038/s41598-025-94323-4 (PMC11937271; doi:10.1038/s41598-025-94323-4)
Supplement: Supplementary file 1 — Supplementary Material 1 [file 41598_2025_94323_MOESM1_ESM.pdf]

Nightlife and low immunity drove transmission of SARS-CoV-2 Gamma in Luxembourg, 2021

Yolanda Pires Afonso[1, *], Dritan Bejko[1], Corinna Ernst[1], Conny Huberty[1], Anke Wienecke-Baldacchino[2], Sibel Berger[2], Malte Herold[3], Cécile Walczak[3], Leslie Ogorzaly[3], Anne Vergison[1], Joël Mossong[1]

Supplementary information

Phases of the public COVID-19 vaccination campaign rollout

The Luxembourg vaccination rollout started on 28 December 2020 (17). To determine eligibility for vaccination, general practitioners issued certificates verifying the patient's vulnerability status. The vaccination campaign was structured into six phases according to the state of vulnerability and risk factors, prioritizing those at the highest risk, and gradually expanding to include other groups of the population (18).

Phase 1 prioritized hospital and healthcare staff, retirement and care home residents, and individuals with disabilities in specialized facilities. Phase 2 focused on highly vulnerable individuals, including those over 75, with trisomy 21, organ transplant recipients, and cancer patients under active treatment, as well as those with congenital immune deficiencies. Phase 3 targeted significantly vulnerable individuals aged 70 to 74, those with acquired immunodeficiencies, severe chronic respiratory or cardiovascular diseases, major neurocognitive deficits, chronic renal failure on dialysis, stage B or C cirrhosis, and morbid obesity. Phase 4 focused on moderately vulnerable individuals aged 65 to 69, including those with diabetes with complications, complicated hypertension, and neuromuscular diseases with significant impact. Phase 5 broadened to include individuals aged 55 to 64 with balanced diabetes, uncomplicated hypertension, and obesity. Lastly, Phase 6 included the general resident population aged 16 to 54, prioritizing the oldest and those not previously eligible due to vulnerability.

As of 13 June 2021 (week 23), 40.4% of Luxembourg's population had received at least one dose, and 28.2% had completed vaccination (19).

Pandemic context of public health measures in the hospitality sector

On June 13, 2021, the Luxembourgish government gradually lifted COVID-19 restrictions by introducing digital COVID-19 certificates (DCCs) in the hospitality sector, called the CovidCheck regime. Prior to this in spring 2021, while the general public was no longer under curfew, the hospitality industry had been limited to operate between 6 a.m. to 10 p.m. with strict seating limitations per table indoors (a maximum of four customers per table) and outdoors and masking when leaving the table. As

from June 13, bars and restaurants were able to resume their usual opening hours (i.e., 1 a.m. or later for nightclubs subject to certain conditions) and the seating restrictions were adapted.

Bars and restaurants could choose between operating under the CovidCheck regime or without it. Under the CovidCheck regime, customers and staff could be inside without a mask if they were fully vaccinated, had a negative PCR test result within 72 hours or a certified negative antigen test within 24 hours, or had a recovery from COVID-19 certificate. Initially, establishments could also provide rapid antigen tests to their customers prior to entry, although this option was removed at a later date. If the CovidCheck regime was not implemented, indoor seating was limited to four individuals per table and outdoor seating to ten individuals per table. Customers had to consume food or drinks while seated at the table. A physical separation of tables by 1.5 meters or the use of Plexiglas barriers was also required. Masks had to be worn by customers when not seated and by staff when in direct contact with customers. Due to the much lower restrictions for customers and staff, almost all bars and restaurants chose to operate under the CovidCheck regime.

Supplementary Figures

Fig. S1

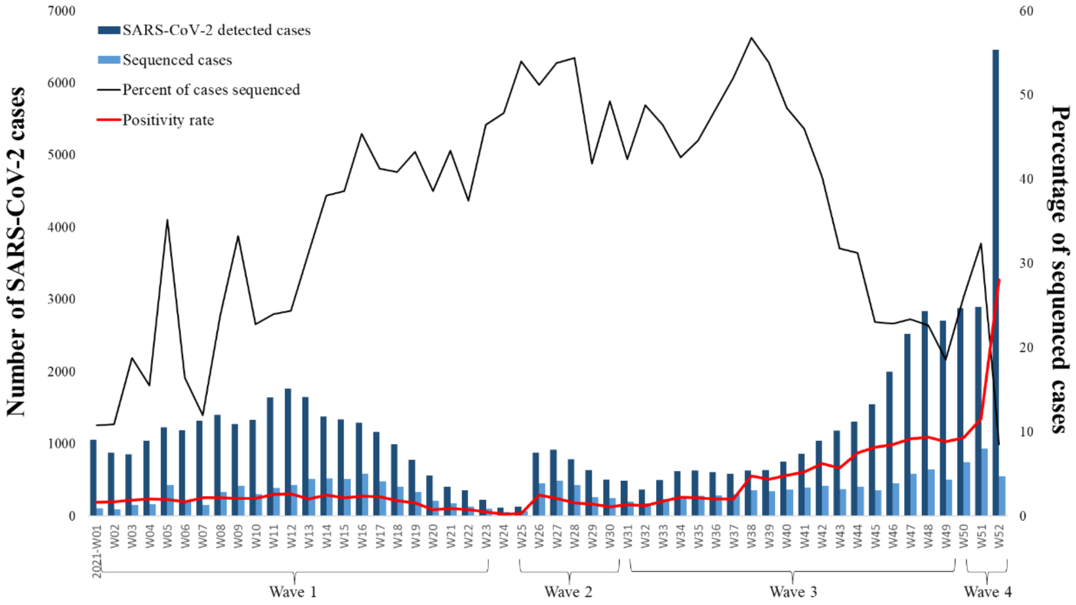

Epidemic curve of SARS-CoV-2 detected cases by week in 2021 by the Luxembourg national surveillance system. Temporal distribution of the number of SARS-CoV-2 detected cases (dark blue bars), percentage of sequenced cases (light blue bars), percentage of cases sequenced (black line), and positivity rate (red line) per week in Luxembourg.

Fig. S2

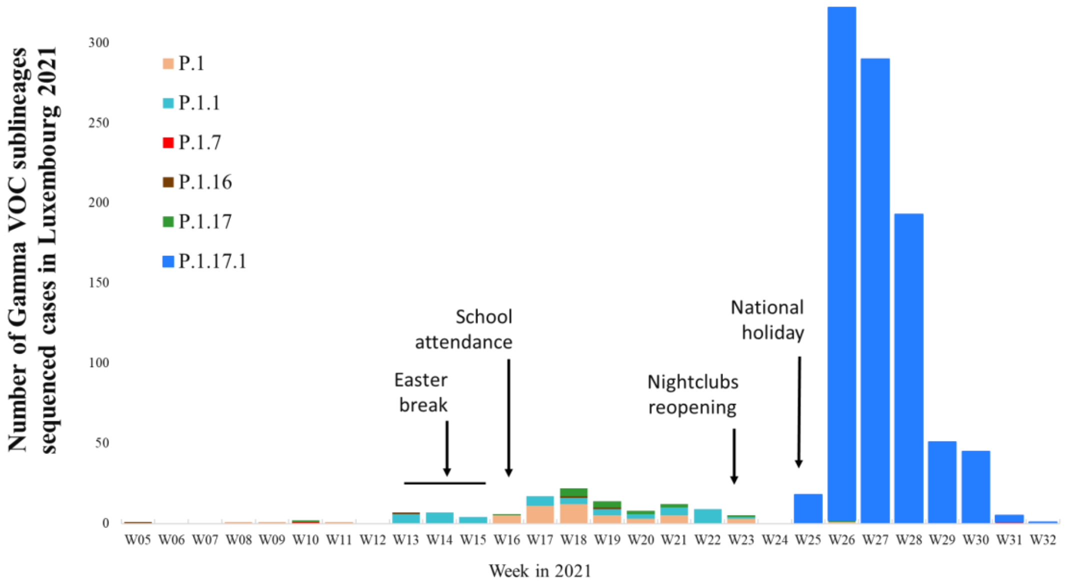

Genomic identification of SARS-CoV-2 Gamma sublineages between February and August (week 05-32) in Luxembourg 2021 as per PANGO classification. Weekly distribution of SARS-CoV-2 Gamma sublineages (P.1, P.1.1, P.1.7, P.1.16, P.1.17 and P.1.17.1) in Luxembourg through the year of 2021. Key events are highlighted with black arrows.

Fig. S3

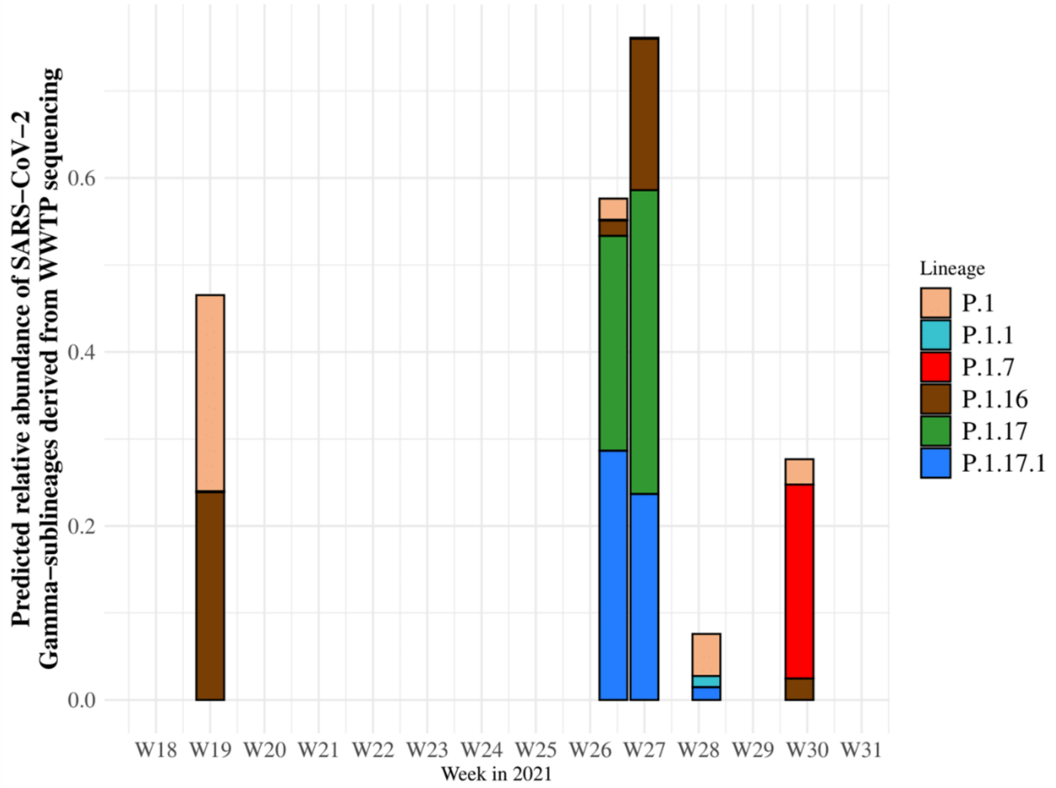

Sublineage abundances predicted from whole genome sequencing of wastewater samples. Relative abundances of Gamma sublineages are shown during the timeframe 2021-05-01 to 2021-07-31 across 3 WWTPs (BEG = Beggen, SCH = Schifflange, PET = Petange). Abundances were predicted directly from sequencing reads with a reference-based method (VLQ). All sequenced samples that passed quality control criteria (average depth > 10, coverage > 40%) are shown.

Fig. S4

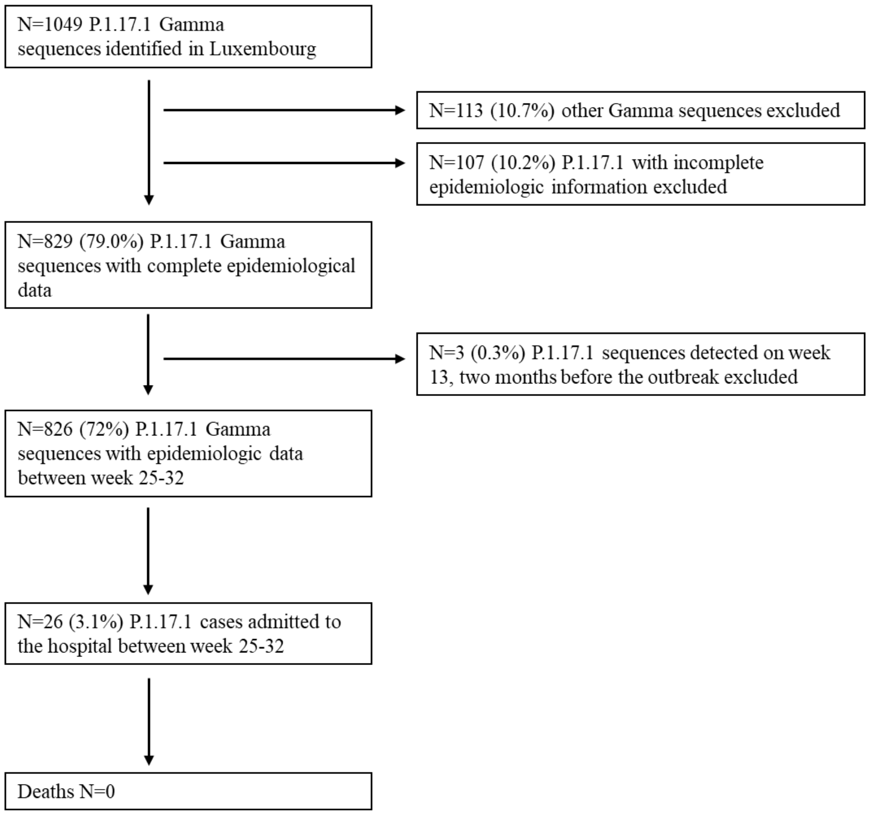

Flowchart depicting the SARS-CoV-2 Gamma VOC study design. The illustration depicts the progression and exclusion criteria applied in the current study period. Out of 1,049 sequences, 829 were laboratory-confirmed as P.1.17.1 strain and had complete epidemiological data; of these, 826 cases correspond to the Gamma outbreak period. Among these, 26 cases resulted in hospitalization, with no reported deaths. Cases excluded comprise other gamma sequences, those with incomplete epidemiological information, and detection that preceded the outbreak.

Fig. S5

a

Gene	Mutation (aa)	P.1	P.1.17.1	P.1.17.1 - C26645T
N	P80R			
	S201S			
	T198I			
	RG203KR			
ORF1b	P314L			
ORF3a	S253P			
	I118V			
	I118M			
	V202L			
ORF6	P57L			
	E55			
ORF8	E92K			
	H28R			
	E19			
S	D138Y			
	D614G			
	E484K			
	H655Y			
	K417T			
	L18F			
	N501Y			
	P26S			
	R190S			
	T1027I			
	T20N			
	V1176F			
	S813N			
	N1119N			
	T618T			
	P681H			
	L216L			
	D936Y			
	H49Y			
M	N41N			

b

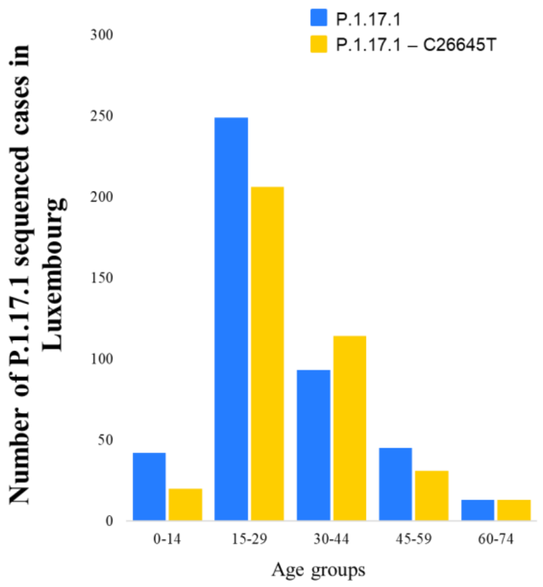

P.1.17.1 Gamma mutational signature and relative distribution in Luxembourg per age group. (A) Key defining mutations are highlighted for SARS-CoV-2 Gamma (P.1) variant (grey) and for P.1.17.1 and P.1.17.1-C26645T strains (blue and yellow, respectively). **(B)** Relative distribution of P.1.17.1 and P.1.17.1-C26645T strains according to age groups among SARS-CoV-2 confirmed cases in Luxembourg.

Fig. S6

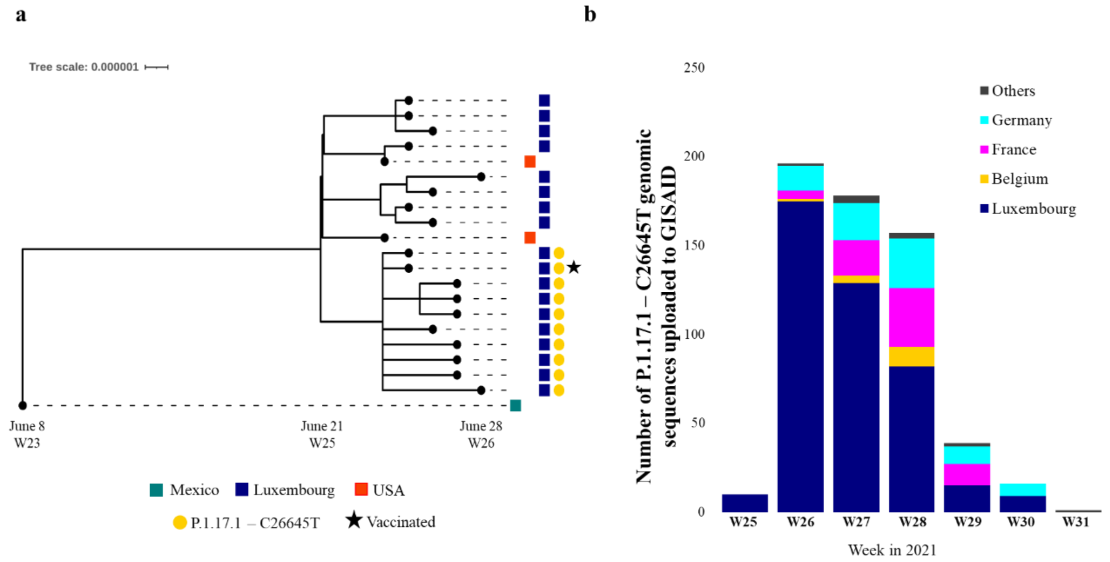

Emergence and global distribution of the VOC Gamma P.1.17.1 sublineage. (A) Gamma P.1.17.1 phylogenetic relationships between the earliest detected genomes from the USA and Mexico and their close relation to the first genomes identified in Luxembourg. The scale bar in the phylogenetic tree indicates the substitutions per site that corresponds to the length of branches in the tree. **(B)** Epidemic curve depicting the total number of P.1.17.1-C26645T genomic sequences (N = 597) from Luxembourg (N = 421), Belgium (N = 16), France (N = 72), Germany (N = 80) and other European countries (N = 11, e.g., Lithuania, Spain, Canary Islands, Northern Ireland, Denmark, Poland, Switzerland, Switzerland) by date of sample collection (weeks) retrieved from GISAID.

Table S1 – Descriptive analysis of cases characteristics of the P.1.17.1 outbreak in Luxembourg, 22 June–15 August 2022 (N = 826 cases).

	**Full sample**						**Sub-sample**					
	**Outbreak phase**				**Total**		**Outbreak phase**				**Total**	
	**W25 – W26**		**W27 – W32**				**W25 – W26**		**W27 – W32**			
**Categorical variables**	N	%	N	%	N	%	N	%	N	%	N	%
Sample size	326	39.5%	500	60.5%	826	100.0%	157	75.8%	50	24.2%	207	100.0%
Source of infection												
Family	17	5.2%	152	30.4%	169	20.5%	2	1.3%	0	0.0%	2	1.0%
Nightlife	160	49.1%	83	16.6%	243	29.4%	141	89.8%	50	100.0%	191	92.3%
Education	12	3.7%	35	7.0%	47	5.7%	0	0.0%	0	0.0%	0	0.0%
Workplace	17	5.2%	40	8.0%	57	6.9%	3	1.9%	0	0.0%	3	1.4%
Travel	5	1.5%	12	2.4%	17	2.1%	0	0.0%	0	0.0%	0	0.0%
Other	1	0.3%	10	2.0%	11	1.3%	0	0.0%	0	0.0%	0	0.0%
Undetermined	114	35.0%	168	33.6%	282	34.1%	11	7.0%	0	0.0%	11	5.3%
Sex												
Female	160	49.1%	233	46.6%	393	47.6%	78	49.7%	23	46.0%	101	48.8%
Male	166	50.9%	267	53.4%	433	52.4%	79	50.3%	27	54.0%	106	51.2%
Age groups												
0-14	9	2.8%	53	10.6%	62	7.5%	0	0.0%	0	0.0%	0	0.0%
15-29	214	65.6%	241	48.2%	455	55.1%	111	70.7%	38	76.0%	149	72.0%
30-44	87	26.7%	120	24.0%	207	25.1%	41	26.1%	9	18.0%	50	24.2%
45-59	14	4.3%	62	12.4%	76	9.2%	5	3.2%	3	6.0%	8	3.9%
>60	2	0.6%	24	4.8%	26	3.1%	0	0.0%	0	0.0%	0	0.0%
C26645T mutation												
No	147	45.1%	295	59.0%	442	53.5%	59	37.6%	30	60.0%	89	43.0%
Yes	179	54.9%	205	41.0%	384	46.5%	98	62.4%	20	40.0%	118	57.0%
Vaccination status												
Not vaccinated	221	67.8%	321	64.2%	542	65.6%	104	66.2%	25	50.0%	129	62.3%
Partially vaccinated	104	31.9%	154	30.8%	258	31.2%	53	33.8%	24	48.0%	77	37.2%
Fully vaccinated	1	0.3%	25	5.0%	26	3.1%	0	0.0%	1	2.0%	1	0.5%
Hospitalisation												
No	322	98.8%	478	95.6%	800	96.9%	155	98.7%	49	98.0%	204	98.6%
Yes	4	1.2%	22	4.4%	26	3.1%	2	1.3%	1	2.0%	3	1.4%
Presence in nightclub												
None							59	37.6%	17	34.0%	76	36.7%
Nightclub 1							24	15.3%	13	26.0%	37	17.9%
Nightclub 2							46	29.3%	4	8.0%	50	24.2%
Other nightclubs							35	22.3%	16	32.0%	51	24.6%
National holidays festivities												
No							44	28.0%	49	98.0%	93	44.9%
Yes							113	72.0%	1	2.0%	114	55.1%
**Continuous variables**	Mean	SD	Mean	SD	Mean	SD	Mean	SD	Mean	SD	Mean	SD
Age	28.4	8.95	29.8	15.5	29.3	13.3	28.4	6.84	27.2	7.7	28.1	7.1

Table S2 – Logistic regression model investigating the likelihood of hospitalization across the outbreak phases, controlling for sex, age, presence of the C26645T mutation and the vaccination status.

	**Outcome: hospitalisation**	
	**OR**	**CI**
Intercept	0.00***	0.00-0.00
Outbreak phase: W27-W32	1.96	0.62-6.20
Sex: Male	2.70*	1.05-6.95
Age (cont.)	1.10***	1.07-1.13
C26645T mutation: Yes	0.52	0.21-1.30
Vaccination status: Fully vaccinated	0.20	0.04-1.03
Vaccination status: Partially vaccinated	0.50	0.17-1.47

Table S3 – Logistic regression model investigating the C26645T mutation as a result of attending specific nightclubs, controlling for sex, age, vaccination status and presence at the national holidays.

	**Outcome: mutation**	
	**OR**	**CI**
Intercept	0.52	0.11-2.46
Nightclub 1	0.12***	0.04-0.34
Nightclub 2	9.99***	3.28-30.41
Other nightclubs	1.39	0.65-2.99
Sex: Male	1.53	0.78-3.00
Age (cont.)	1.01	0.97-1.07
Vaccination status: Fully/partially vaccinated	0.58	0.29-1.16
National holiday festivities: Yes	1.96	0.99-3.89

References

European Centre for Disease Prevention and Control. (2023). *COVID-19 Vaccine Tracker*. https://vaccinetracker.ecdc.europa.eu/public/extensions/COVID-19/vaccine-tracker.html#national-ref-tab

Luxembourg: Vaccination will be carried out in 6 phases. (2021). https://lequotidien.lu/politique-societe/luxembourg-la-vaccination-se-fera-en-6-phases/

Premières vaccinations du personnel des structures d'hébergement pour personnes âgées et des réseaux d'aide et de soins contre la COVID-19 au Luxembourg. (2020). https://gouvernement.lu/fr/actualites/toutes_actualites/communiques/2020/12-decembre/30-vaccinations-personnel-structuresageesetsoins.html
